# Nutrition interventions to treat low muscle mass in cancer

**DOI:** 10.1002/jcsm.12525

**Published:** 2020-01-08

**Authors:** Carla M. Prado, Sarah A. Purcell, Alessandro Laviano

**Affiliations:** ^1^ Human Nutrition Research Unit, Department of Agricultural, Food and Nutritional Science University of Alberta Edmonton Alberta Canada; ^2^ Division of Endocrinology, Metabolism, and Diabetes, and Division of Nutrition, School of Medicine University of Colorado Aurora CO USA; ^3^ Department of Translational and Precision Medicine La Sapienza University Rome Italy

**Keywords:** Low muscle mass, Myopenia, Sarcopenia, Cancer, Nutrition, Protein, Intervention, Body composition

## Abstract

Many patients with cancer experience poor nutritional status, which detrimentally impacts clinical outcomes. Poor nutritional status in cancer is primarily manifested by severe muscle mass (MM) depletion, which may occur at any stage (from curative to palliative) and often co‐exists with obesity. The objective of this article was to discuss gaps and opportunities related to the role of nutrition in preventing and reversing low MM in cancer. It also provides a narrative review of relevant nutritional interventions for patients capable of oral intake. The impact of nutrition interventions to prevent/treat low MM in cancer is not well understood, potentially due to the limited number of studies and of clinically viable, accurate body composition assessment tools. Additionally, the type of study designs, inclusion criteria, length of intervention, and choice of nutritional strategies have not been optimal, likely underestimating the anabolic potential of nutrition interventions. Nutrition studies are also often of short duration, and interventions that adapt to the metabolic and behavioural changes during the clinical journey are needed. We discuss energy requirements (25–30 kcal/kg/day) and interventions of protein (1.0–1.5 g/kg/day), branched‐chain amino acids (leucine: 2–4 g/day), β‐hydroxy β‐methylbutyrate (3 g/day), glutamine (0.3 g/kg/day), carnitine (4–6 g/day), creatine (5 g/day), fish oil/eicosapentanoic acid (2.0–2.2 g/day EPA and 1.5 g/day DHA), vitamin/minerals (e.g. vitamin D: 600–800 international units per day), and multimodal approaches (nutrition, exercise, and pharmaceutical) to countermeasure low MM in cancer. Although the evidence is variable by modality type, interventions were generally not specifically studied in the context of cancer. Understanding patients' nutritional requirements could lead to targeted prescriptions to prevent or attenuate low MM in cancer, with the overall aim of minimizing muscle loss during anti‐cancer therapy and maximizing muscle anabolism during recovery. It is anticipated that this will, in turn, improve overall health and prognostication including tolerance to treatment and survival. However, oncology‐specific interventions with more robust study designs are needed to facilitate these goals.

## Introduction

Cancer is a disease affecting millions of people and one of the leading causes of death worldwide. Proper nutrition can alleviate symptom burden, improve health across the cancer continuum, and support cancer survivorship^1^ and is a hallmark of successful cancer treatment.^2^

The primary nutritional problem experienced by people with cancer—and likely the most impactful on prognosis—is muscle wasting (also termed sarcopenia or myopenia). Low muscle mass (MM) is common regardless of cancer stage (from curative to palliative) and is an independent predictor of poor physical function, lower quality of life, surgical complications, cancer progression, and reduced survival^3^, ^4^, ^5^, ^6^, ^7^. The overall prevalence of low MM is >50% in people with newly diagnosed cancer, which is considerably higher than the approximately 15% prevalence in healthy individuals of similar age (median 65 years old).^8^ This suggests that low MM contributes to the cancer process, either directly or indirectly. Further, as only about 10% of patients are underweight, this widespread phenomenon of low MM occurs independently of body weight or fat mass.^9^

Reversing low MM has the potential to improve cancer therapy outcomes, morbidities, and, ultimately, mortality. Given the role of MM and adipose tissue in oncological outcomes, strategies to optimize body composition are an important part of successful cancer therapy. Nutrition is one such modality that can favourably influence MM and adipose tissue. Herein, we provide a narrative review of the current state of the literature in this area, present an overview of nutrition strategies aimed at enhancing MM, and discuss strategies to mitigate limitations of nutrition interventions.

## Background

Low MM in cancer has been primarily studied under the auspices of refractory cancer cachexia, which is irreversible and non‐responsive to nutrition intervention.^10^ Therefore, skepticism exists in regard to the impact of nutrition interventions to countermeasure MM loss. While both conditions are associated with muscle depletion, low MM in cancer occurs independent of weight loss and therefore of cachexia. Notably, cachexia is also not frequent in all cancer types, while MM loss may occur more frequently, across cancer types, and in the presence of weight maintenance or weight gain.^11^, ^12^ Importantly, patients without refractory cachexia have anabolic potential and can present with gains in MM.^13^ Low MM in cancer also occurs at any disease stage.^14^, ^15^ Many believe that nutrition is not a priority once cancer is diagnosed or even that it can increase tumour growth,^16^ which is not founded on scientific evidence.^17^, ^18^, ^19^

The limited awareness of the impact of nutrition interventions on cancer outcomes can also be related to the (non‐exclusive) following issues:

- Limited use of accurate body composition tools: Although body weight and weight loss can be useful prognostic tools, anthropometric variables do not provide information on body composition alterations; in fact, BMI and BMI loss do not consistently relate to immediate clinical outcomes such as treatment toxicities.^20^ Utilization of sophisticated measurements of body composition in oncology is recent, with the first published study assessing MM using computerized tomography in cancer published in 2007.^7^ The use of accurate and reliable tools such as computerized tomography can not only quantify the impact of an intervention on the amount of MM but can also detect muscle attenuation, which is expressed in Hounsfield units and reflective of intramuscular adiposity (‘quality’ of the muscle). Furthermore, patients with cancer may also have increased skeletal muscle collagen content, which is associated with poor survival^21^; such parameters would not be detected by simple anthropometric assessments. Due to the historically limited availability of body composition measurements, the impact of nutrition interventions to reverse MM loss has been primarily focused on gains in total body weight, without a clear understanding of specific changes in muscle versus adipose tissue compartments.
- Different criteria for low MM: The level of MM below which people are diagnosed with ‘low MM’ remains a contentious topic.^22^ As discussed elsewhere,^23^ cut‐points depend on the methodological assessment, as well as the population and/or outcome of interest. Newer studies have also proposed sex‐specific percentiles and means and standard deviations for skeletal MM based on healthy adult populations.^24^, ^25^
- Funding for nutrition research and small number of randomized controlled trials: Nutrition research receives a small proportion of funding,^26^ especially in comparison with pharmaceutical trials. This has reflected poorly on the funds available to conduct well‐designed (and costly) randomized controlled trials,^27^ which impact guideline recommendations and likely the trust of health care professionals across different disciplines.
- In the absence of anabolic medication, it could take months to completely restore muscle lost over a short period of time.Length of intervention insufficient to impact/measure muscle change: Short‐term interventions may not detect an anabolic response as demonstrated by improvements in MM. In oncology, MM loss may occur rapidly in some individuals and is especially pronounced in metastatic disease, wherein a 6.1% [95% confidence interval (CI) −8.4, −3.8, *P* < 0.001] MM decrease over 3 months has been reported, corresponding to 1.7 kg loss in male patients and 1.1 kg loss in female patients with advanced colorectal cancer.^28^ Even in patients with earlier stage cancer, MM loss ≥5.7% over median 14.3 months has been reported in approximately 20% of individuals (*n* = 1924).^12^ Conversely, regaining MM through behavioural interventions is a much lengthier process. For example, 12 months of structured resistance training results in an increase of <1 kg of lean mass (compartment that includes MM) in breast cancer survivors.^29^, ^30^
  In the absence of anabolic medication, it could take months to completely restore muscle lost over a short period of time. This is analogous to a wildfire, where preserving is better than rebuilding. Therefore, short‐term interventions are unlikely to have a meaningful impact on patient prognostication. As shown by several cross‐sectional studies, patients present with muscle depletion at the time of cancer diagnosis and continue to lose muscle throughout the disease trajectory, even in the context of curative disease.^4^, ^9^, ^12^, ^14^ Therefore, a nutrition intervention that can halt muscle wasting or functional decline (i.e. delta muscle change = zero) can be considered a positive outcome.^31^, ^32^ This is supported by internationally accepted European Society for Clinical Nutrition and Metabolism (ESPEN) guidelines on nutrition in cancer, which state that the ‘*goals of nutritional and metabolic therapy, therefore, must place considerable emphasis on maintenance or gain of muscle mass*’.^18^ Furthermore, nutrition intervention should be life‐long, aimed at minimizing MM loss during catabolic circumstances (i.e. anti‐cancer treatment) and maximizing MM anabolism during recovery. Nutrition strategies should also adapt to the metabolic and behavioural changes occurring during the clinical journey. This time span is not captured within the current research landscape.
- Inclusion criteria limited to patients with short life expectancy: As mentioned previously, the impact of nutrition interventions to halt or reverse muscle loss has been primarily studied in the context of cachexia, and many were published before our understanding of limited anabolic potential of patients with refractory cachexia.^10^, ^13^ As such, the inclusion criteria of many clinical trials is composed of patients with refractory cachexia, precluding a positive outcome. Even though protein synthesis of these patients can be acutely stimulated by targeted nutrition intervention,^33^ the few weeks/months life expectancy would likely prevent meaningful changes in MM and, in turn, an impact on patient prognostication. Therefore, the inclusion criteria of a number of studies where patients had to be within 3 months life expectancy or less^34^, ^35^—likely having refractory cachexia^10^—impede the evaluation of the true impact of a nutrition intervention.
- Limited understanding of effective nutrition strategies to stimulate MM anabolism alone and in combination with exercise and other complementary therapies (multimodal approaches). While multimodal interventions may be effective at maintaining MM in cancer,^36^ the efficacy of individual components of such interventions has been scarcely characterized. A better understanding of specific nutrients' contributions to muscle anabolism in these patients can lead to the development of specialized nutritional products focusing on halting MM in cancer.

In view of the information above, our understanding of optimal nutrition interventions to countermeasure low MM in cancer is at its infancy. Given the importance of nutrition in oncology, describing the current research landscape within this area is imperative to designing well‐informed research and clinical pathways aimed at mitigating MM loss in cancer.

## Nutrition intervention in cancer: gaps and opportunities

**Figure 1:**
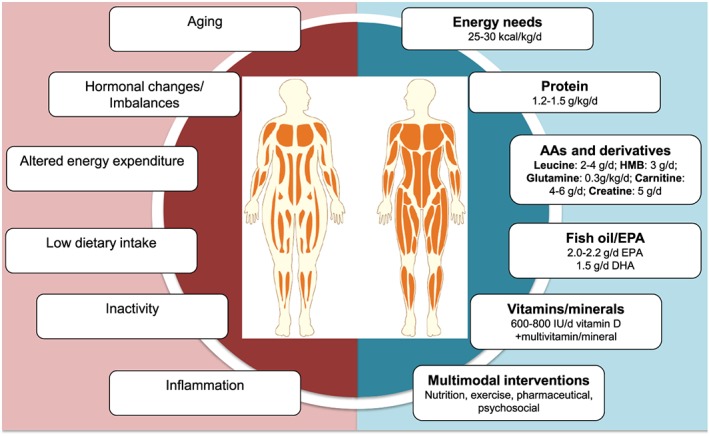
Causes and potential nutrition treatments for low muscle mass in cancer. Items on the left negatively impact muscle mass, while items on the right are under consideration for treating low muscle mass. Amounts on the right are either current recommendations (where available) or what research suggests might be sufficient for altering body composition. Interventions are focused on patients capable of oral nutrition intake. AAs, amino acids; DHA, docosahexaenoic acid; EPA, eicosapentanoic acid; HMB, β‐hydroxy β‐methylbutyrate; IU, international units.

Nutrients under consideration for the treatment of low MM in cancer are shown in *Figure* 1. We performed a literature search in PubMed from inception until 25 April 2019. The search strategy consisted of terms referring to nutrition intervention, low MM/sarcopenia, and cancer; key words within each component were linked using ‘OR’ as a Boolean function, and the results of the three components were combined by utilizing the ‘AND’ Boolean function. We limited our review to articles published in English, human adults, and those that measured body composition using bioelectrical impedance analysis, dual X‐ray absorptiometry, computerized tomography, air displacement plethysmography, or magnetic resonance imaging. Only clinical trials, case reports, journal articles, or observational studies were included in the search. A total of 2791 publications were initially uncovered; relevant articles are discussed below.

### Energy

Determining energy requirements of patients with cancer is a challenge.^37^ Optimal levels of energy intake are needed not only to avoid weight loss but also to maintain MM by stimulating protein synthesis and suppressing protein breakdown. Cancer‐related weight loss has long been understood as leading to muscle loss (‘the skeleton in the closet’).^38^, ^39^ However, clinical implications of gains in adipose tissue versus that of MM are different. Muscle is an important tissue for movement and mobility, balance, posture, and strength, in addition to being a reservoir of amino acids and site of myokine production. While adipose tissue is still important,^40^ excessive adiposity may not confer a survival advantage, especially in the context of low MM.^4^, ^41^ Previous observations of the obesity paradox (i.e. overweight or obesity leading to favourable outcomes) in cancer may be due to the lack of body composition data and the use of body mass index, which confounds the association of low MM with shorter survival.^14^, ^42^ As such, interventions leading to disproportional gains in adipose tissue versus MM might have limited impact on clinical outcomes or its specific contribution to reversing low MM. Characterizing energy requirements through the accurate determination of energy expenditure is therefore a fundamental step in the nutrition care process.

Most of our understanding of energy requirements in cancer refers to studies assessing resting energy expenditure (REE), which is the largest and most commonly measured component of total energy expenditure. While most healthy adults have a measured REE that is within 10% of its predicted value, a substantial proportion of patients may present with an REE that falls outside of this range.^37^, ^43^ Furthermore, REE might change throughout the disease trajectory and is impacted by systemic inflammation and cancer stage.^44^, ^45^ Unfortunately, total energy expenditure has been studied in only five small cancer cohorts using doubly labelled water or bicarbonate urea methods and primarily in patients with advanced stages of cancer.^46^, ^47^, ^48^, ^49^, ^50^ Total energy expenditure appears to be lower in patients with pancreatic cancer despite high REE (signifying low physical activity),^51^ although others have reported that predicted REE expressed as a percent of measured does not relate to total energy expenditure.^47^ In patients with primarily early‐stage colorectal cancer, total energy expenditure differs according to body mass, body composition, and physical activity but is not related to REE expressed as a percent of predicted REE (i.e. how ‘hypermetabolism’ is typically detected).^50^ More studies of total energy expenditure are required to elucidate energy requirements in this population.

**Figure 2:**
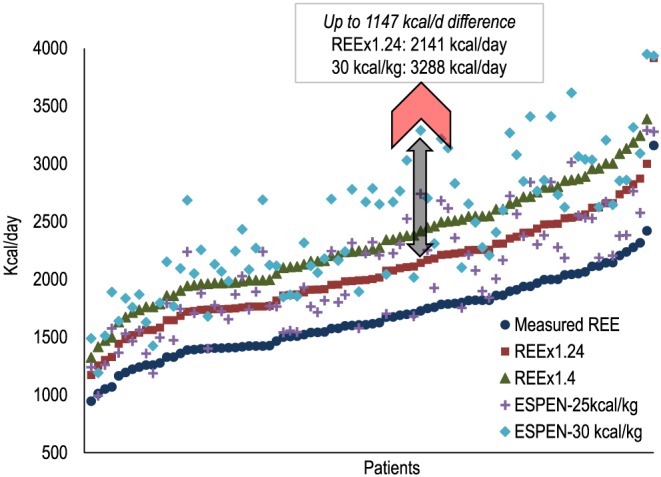
Comparison of theoretical energy recommendations among patients with colorectal cancer. *n* = 83 patients with newly diagnosed stage I–IV colorectal cancer had resting energy expenditure (REE) measured by indirect calorimetry (metabolic cart). Each quintuplicate of shapes represents one patient. REE was multiplied by a physical activity level of 1.24 and 1.4 according to previous research in cancer^50^ and energy recommendations put forth by FAO/WHO/UNU for adults,^51^ respectively. Energy requirements were also estimated by multiplying body weight (in kg) by 25 and 30 as per oncology‐specific recommendation put forth by The European Society for Clinical Nutrition and Metabolism (ESPEN).^18^

Because of the paucity of data measuring total energy expenditure, current oncology energy recommendations put forth by ESPEN are based on body weight alone (25–30 kcal/kg/day).^18^ Other methods of estimation include estimating or measuring REE and multiplying by an assumed physical activity level.^52^ These methods, however, likely produce different estimations of energy requirements, further complicating energy recommendations. As a theoretical exercise to investigate the heterogeneity of such recommendations, we estimated energy requirements using methods that might be commonly utilized in clinical practice. A sample of 83 individuals with newly diagnosed stage I–IV colorectal cancer had REE measured by indirect calorimetry (Vmax 29N, Yorba Linda, CA, USA) as previously described.^53^ In each patient, a physical activity level of 1.24 was chosen to estimate total energy expenditure from measured REE according to previous literature^51^ and another physical activity level of 1.4 was used to estimate energy requirements according to the minimum amount considered ‘sedentary’ by the Food and Agricultural Organization of the United Nations/World Health Organization/United Nations University.^52^ Energy requirements were also estimated as 25–30 kcal/kg/day, in line with current oncology‐specific guidelines.^18^ As shown in *Figure* 2, these calculations produced highly discrepant estimations of energy requirements in each individual. In fact, differences were up to 1147 kcal/day, and 52 (62.7%) patients had ≥500 kcal/day discrepancy between at least one pair of energy requirement recommendations.

The issue of inaccurate estimation of energy requirements directly and substantially impacts dietary prescriptions and hence our ability to optimize patient's nutritional status. Patients with low MM and obesity (sarcopenic obesity) pose an additional challenge, because these individuals might benefit from a caloric controlled diet with high protein content. However, without a thorough understanding of total energy expenditure, nutritional interventions are based on estimation of body weight which may exacerbate MM loss or increase fat mass. We posit that changes in body composition, tumour burden, systemic inflammation, brown adipose tissue activation, physical activity, dietary intake, and treatment modalities may all affect energy expenditure in cancer, contributing to the variability between actual energy requirements and current unspecific recommendations.^54^ Therefore, improved characterization of total energy expenditure in relation to these factors and across the disease trajectory is needed to define evidence‐based energy recommendations.

### Protein

Although single nutrients and investigational drugs are under consideration for treating low MM in cancer, an adequate supply of protein is the foundation for maintenance or gain of muscle. Net muscle protein balance is required for increasing skeletal MM, and nutrition is a potent anabolic stimulus. Specifically, the postprandial increase in circulating amino acids stimulates muscle protein synthesis.^55^ Any other treatment for low MM in cancer may fail without an adequate quantity and quality of protein.^23^ Optimal amounts of protein for preventing or treating low MM in cancer are undefined; presently, we do not know the definition of ‘adequate protein’ and if this amount differs in various patient groups. We hope that upcoming studies using indicator amino acid oxidation method and randomized clinical trials exploring the impact of escalating protein doses on MM will provide the much‐needed groundwork to begin to define protein requirements (e.g. http://Clinicaltrials.gov 56).

Guidelines on protein intake in cancer are not directed at low MM, being given as a range of 1.0–1.5 g/kg/day.^18^ There is little evidence of guideline efficacy and safety (assuming no kidney failure is present). Further, many patients with cancer do not meet this standard or even the guideline for healthy individuals (0.8 g/kg).^57^ Reported intakes in the literature range widely from 0.2 to 2.7 g/kg.^58^, ^59^ We previously reported that many (35%) people with advanced lung or colorectal cancer consumed <1.0 g/kg/day and that protein intake positively related to MM (*r* = 0.40, *P* = 0.001).^60^ Others have shown that only essential amino acids matter for protein anabolism, highlighting the importance of high‐quality protein diets.^61^ Recently, we explored current guidelines that adjust protein intake by body weight.^23^, ^62^, ^63^ This particular practice ignores the large variability of body composition in contemporary populations.^4^ As amino acids stimulate muscle protein synthesis and provide the building blocks for anabolism, protein recommendation based on muscularity is biologically logical. While it is premature to adjust protein needs by body composition and lean mass, this may be a targeted approach to optimize individual protein requirements.

Limited protein intake may be due to nutrition impact symptoms such as anorexia, taste/smell alterations, dysphagia, nausea, vomiting, which are prevalent in some cancer types and might negatively affect dietary intake, especially in advanced cancer.^64^ Furthermore, individuals with cancer might purposely alter their diet, as diagnosis may be a major source of motivation in making lifestyle changes.^65^ However, without adequate dietary guidelines, many individuals might avoid animal products (particularly red meat^66^), although the net effect on total protein intake has not been documented. Interestingly, red meat intake was *inversely* associated with 7‐year mortality among 992 individuals with stage III colon cancer,^67^ which contradicts current guidelines.^68^ These findings suggest that higher protein intake is beneficial in oncology populations, although further research is needed to elucidate the appropriate amount and type of protein needed to support overall health and survivorship. Among patients with active disease, a review of cancer cachexia studies concluded that dietary protein >1.5 g/kg/day can maintain or improve MM, and these effects might be more substantial when combined with exercise in other clinical populations with cachexia.^69^ This is also in line with the recently published position paper from the Society of Sarcopenia, Cachexia and Wasting Disorders.^70^ A general protein recommendation for elderly individuals is to consume 1.5 g/kg/day or about 15–20% of total caloric intake to prevent low MM and optimize function.^71^ In the context of low MM in cancer, it is still unknown whether 1.5 g/kg/day is a sufficient amount to favourably modulate body composition.

When writing the new oncology nutrition guidelines, ESPEN noted the lack of current studies to update previous recommendations.^18^ This recently released guideline suggests that a higher range of protein intake (1.2–1.5 g/kg/day) is needed and that 2.0 g/kg/day is associated with positive protein balance, according to their literature review, although research is needed to support the latter as a recommendation.^18^ In fact, experts authoring this guideline encouraged the development of evidence‐based studies to substantiate and compare the recommended amounts. Additionally, more research is needed on the clinical effect and feasibility of high protein intake and the optimal composition of amino acids. The timing of protein intake may also impact muscle protein synthesis, because a balanced distribution of protein (i.e. approximately equal amounts consumed throughout the day) more favourably enhances 24 h muscle protein synthesis compared with unbalanced protein distribution in young adults.^72^ Notably, however, most studies investigating protein and amino acid intake have focused on metabolic endpoints such as protein synthesis, and few have examined changes in body composition in relation to protein consumption and clinical endpoints.^73^

Although it was once believed that muscle anabolism was implausible in cancer, these patients have anabolic potential,^13^, ^33^, ^61^, ^74^ despite their older age, inactivity, systemic inflammation, or insulin resistance. Previously reported anabolic resistance may refer to a higher threshold needed for protein synthesis in response to an anabolic stimulus.^75^, ^76^

### Branched‐chain amino acids

Because of their critical role in promoting protein synthesis in muscle tissue, branched‐chain amino acids (BCAAs; leucine, isoleucine, and valine) have been investigated as a target for nutritional therapy. In patients with advanced intra‐abdominal metastatic adenocarcinoma, higher whole body protein synthesis and leucine balance was observed after a parenteral nutrition infusion of 50% BCAAs compared with a formula with 19% BCAAs.^77^, ^78^ Another study reported that a nutritional supplement with 40 g of casein‐based and whey‐based protein enriched with 4.16 g leucine, fish oil, and oligosaccharides induced a higher muscle protein fractional synthetic rate compared with a standard supplement with 24 g protein in patients with advanced cancer (mixed tumour types).^33^ In healthy elderly subjects, leucine induces protein synthesis in doses >~2 g, but long‐term supplementation does not necessarily equate to increased MM.^79^ Current evidence suggests that BCAAs might help to ameliorate muscle loss in cancer, although future investigations in the efficacy of BCAAs alone in different cancer types and the effects on glucose metabolism^80^ are needed.

### β‐hydroxy β‐methylbutyrate

β‐hydroxy β‐methylbutyrate (HMB) is a metabolite of the BCAA, leucine, and is found naturally in low levels in the diet. It is thought to modulate protein turnover, primarily by minimizing protein degradation.^81^ Proteolysis is the main mechanism by which muscle is lost in cancer^82^; thus, HMB has been investigated as a potentially effective nutritional supplement. To our knowledge, there are no published studies that assessed the effect of HMB alone on cancer‐associated muscle wasting; rather, HMB has been combined with other amino acids in supplements. In patients with advanced cancer (mixed tumour types), a supplement with 3 g HMB, 14 g arginine, and 14 g glutamine induced a significant gain in lean mass, while those randomized to the control supplement without essential amino acids (only non‐essential amino acids) lost lean mass after 4 weeks (1.12 ± 0.68 kg, vs. −1.34 ± 0.78 kg), which was sustained after 24 weeks.^83^ However, a study which used the same supplement in 175 patients with various tumour types and previous weight loss (3 g HMB, 14 g arginine, and 14 g glutamine) for 8 weeks found only a trend towards higher lean mass (*P* = 0.08) in those randomized to the study supplement.^84^ A greater effect of the supplement was noted in patients with smaller initial weight loss (<5%), which suggests that some patients in the study might be in the refractory cachexia stage at which point nutrition interventions are unlikely to provide benefit. In addition, the short follow‐up time of 8 weeks might have precluded significant body composition changes. Another study provided HMB with arginine and glutamine (1.2 g HMB, 7 g arginine, and 7 g glutamine) 3 days before and 7 days after surgery in 60 patients with abdominal malignancies.^85^ No differences in MM was observed between supplement and placebo groups, perhaps due to the short duration of the intervention. One systematic review noted that HMB has a beneficial effect on body composition in other clinical populations and recommended a dose of 3 g/day to improve body composition.^86^ Similarly, a recent systematic review and meta‐analysis found a moderate positive effect of HMB on change in MM among various clinical conditions, although effect size was small (0.25 standard mean difference, 95% CI: −0.00, 0.50, *P* = 0.05).^87^ Given the efficacy of HMB in healthy elderly, HIV/AIDs, chronic obstructive pulmonary disease, and rheumatoid arthritis,^86^, ^87^ HMB could be a potential preventative supplement to countermeasure low MM in cancer, although further research with longer interventions is needed.

### Glutamine

Glutamine is a non‐essential amino acid that has many roles in human metabolism. It is a fuel source for gastrointestinal enterocytes, a substrate for gluconeogenesis, and acts as a nutrient in muscle protein metabolism during infection, inflammation, and trauma.^88^ Glutamine can become conditionally essential in disease states such as cancer. While glutamine has typically been used to ameliorate mucositis and increase the selectivity of anti‐cancer drugs,^89^ it might be useful in preventing low MM due to its role in synthesizing protein. Randomized controlled trials have reported no adverse effects of supplementation.^90^, ^91^ A study in 44 patients undergoing surgery for non‐metastatic head and neck cancer randomized half of the sample to consume glutamine supplementation (0.3 g/kg/day) for 4 weeks versus placebo. All patients in the intervention group increased lean mass post‐operatively, while only one person in the control group did so.^92^ Of note, these findings should be interpreted with caution because the study was small and of short duration; there is not enough evidence to support the general use of glutamine in patients with cancer. Nevertheless, this amino acid should be explored for the prevention/treatment of low MM given its effectiveness in ameliorating treatment side effects and efficacy of improving body composition in the aforementioned trial.

### Carnitine

Carnitine is a di‐peptide which can be obtained from food or formed via the conversion from lysine and has traditionally been used in athletic populations as an ergogenic aid.^93^ Individuals with cancer might have low plasma carnitine levels due to reduced food intake and decreased carnitine absorption and increased urinary excretion as a side effect of certain chemotherapy regimens.^94^ In a randomized double‐blinded trial, 72 patients with advanced pancreatic adenocarcinoma received 4 g carnitine or placebo for 12 weeks.^95^ The authors reported that body cell mass (the ‘homogeneous energy‐exchanging, work‐performing moiety of body tissue’^96^) was significantly higher at the end of the study in the carnitine group (*P* = 0.013, body composition values not reported). Notably, pancreatic cancer is a severe form of disease often characterized by metabolic disturbances and excessive weight loss, precluding universal conclusions about carnitine across cancer types. Another study found that when 12 patients with cancer (mixed tumour types, stages III and IV) consumed 6 g l‐carnitine, lean mass increased over 4 weeks (38.0 ± 7.4 to 40.4 ± 8.6 kg, *P* < 0.05).^97^ Because of the distinct cancer populations and short study durations, there is not enough evidence to recommend carnitine as a potential supplement to prevent or mitigate low MM.

### Creatine

Creatine is a tripeptide composed of arginine, methionine, and glycine. Creatine can be obtained from an omnivorous diet, and it has been used as a supplement for decades in athletic populations due to its ability to improve performance during short, high intensity bouts of activity.^98^ In older populations, creatine supplementation can improve lean mass and muscle function and has been used in dozens of interventions in this population.^99^

Less is known about creatine supplementation in cancer, as recently reviewed in depth.^100^ One double‐blind randomized controlled trial selected 30 individuals with stage III or IV colorectal cancer to receive creatine (4 × 5 g for the first week and then 2 × 2.5 g for 7 weeks) or placebo.^101^ Individuals in the creatine arm had higher phase angle (a measure of cell membrane integrity and nutritional status) and hand grip strength at the end of the study.^101^ Another intervention randomized patients with stage I–IV head and neck cancer to 12 weeks of resistance training with (*n* = 14) or without (*n* = 7) 5 g/day creatine and 30 g/day protein supplements.^102^ The group with creatine increased lean mass by 5.0 ± 3.8% from baseline (*P* < 0.001) while the control group did not significantly increase lean mass (2.8 ± 2.5%, *P* = 0.07). Muscle strength also improved, with no significant differences between groups.^102^ Conversely, another study using bioelectrical impedance analysis in individuals with incurable malignancies found no differences in body composition after 1 month of creatine supplementation (*n* = 20 with body composition measures) versus the control group (*n* = 15).^103^ Notably, all studies consisted of small groups of patients with viable body composition measurement, limiting current understanding of the effectiveness of creatine. Further research is needed to elucidate the potential efficacy of this supplement for mitigating low MM in cancer.

### Fish oil and eicosapentaenoic acid

Eicosapentaenoic acid (EPA) is an anti‐inflammatory substance, and thus, EPA alone or in fish oil supplements has been investigated for use in cancer to preserve skeletal muscle, usually under the auspices of cachexia. There are several uncontrolled, not‐randomized studies that indicate that EPA/fish oil is an effective modality to reduce systemic inflammation, induce weight gain, increase dietary intake, and improve performance status.^104^ The first randomized, double‐blind trial enrolled 200 patients with stage II–IV pancreatic cancer and cachexia to a target consumption of 2.2 g EPA in 620 kcal of supplement.^46^ Non‐compliance in control and experimental groups might have led to an equal degree of weight change in both groups, but correlation analysis revealed a significant association between supplement intake and body weight gain (*r* = 0.50, *P* < 0.001) and increase in lean mass (*r* = 0.33, *P* = 0.036) that was only present in the experimental group. Another study by Murphy *et al*.^32^ randomized patients with stage III–IV lung cancer to receive fish oil (2.2 g EPA/day) or standard‐of‐care. During chemotherapy, approximately 69% of patients taking fish oil maintained or gained skeletal muscle (measured by computed tomography imaging) compared with 29% of patient receiving standard‐of‐care. In addition, four patients (out of 24) in the standard‐of‐care group developed low MM during treatment, while none in the fish oil group developed low MM. Individuals with higher concentrations of plasma EPA after supplementation had the greatest gains in muscle (*r* ^2^ = 0.55, *P* = 0.01). Intramuscular adipose tissue increased by 9.5 ± 5.2 %/100 days in the control group but decreased by 16.4 ± 13.9%/100 days in the fish oil group, which was significantly different. The accumulation of intramuscular adipose tissue has been implicated in insulin resistance and cachexia; fish oil and EPA could therefore prevent the development of metabolic abnormalities, although this merits further investigation.

While several other studies reported clinically and statistically significant effects of fish oil on preventing muscle loss,^105^, ^106^, ^107^, ^108^, ^109^ others have found no effect.^46^, ^110^, ^111^ Such discrepant findings may be due to methodological issues such as patient compliance and cross‐over design. Of note, most outcomes in these publications used anthropometric measures or bioelectrical impedance analysis, which have notable limitations when assessing body composition in cancer, as fluid imbalance is common. In addition, publications usually investigate EPA/fish oil supplementation in advanced cancer or in tumour groups that are very likely to develop cachexia (such as pancreatic). While some authors posit that patients should avoid fish oil due to the potential for chemotherapy resistance,^112^ their conclusions were based on rodent data, which do not provide a sufficient basis for human dietary recommendations.^113^, ^114^ Furthermore, some studies suggest that omega‐3 fatty acids *inhibit* tumour cell proliferation^115^ and might decrease toxicity.^116^

Because of several recent positive clinical trials, a plausible biological rationale, and small side effects, fish oil and EPA could help to improve appetite, food intake, body weight, and MM in individuals at risk for body composition alterations.^18^ Importantly, high protein oral nutrition supplements enriched with EPA may help ameliorate weight and MM loss to a greater extent than isocaloric control supplements.^117^ Given the large number of studies reporting a positive impact on MM, it is likely that fish oil would be a practical and effective modality for preventing muscle loss without significant side effects.

### Vitamins and minerals

Some individuals with cancer will become malnourished during the disease trajectory, and there is an inherent risk of micronutrient deficiency. In addition, side effects of therapy such as vomiting or diarrhea might void the body of micronutrients and decreasing levels of vitamins A and E have been observed during radiation.^118^ Other nutrients of concern include vitamins C, D, and E, some B vitamins, zinc, and selenium.^118^ A recent systemic review concluded that there is insufficient evidence to support the use of any vitamin or mineral supplement.^119^ However, this review included patients with cachexia only (>5% weight loss in previous 6 months, hypermetabolism, and/or reduced food intake), which have a different body composition and metabolic profile than individuals with low MM but without cachexia. Because of the adverse effects of therapy and restricted diet of many patients, the American Institute for Cancer Research,^120^ American Cancer Society,^68^ and ESPEN^18^ support the use of a multivitamin‐multimineral supplement in doses close to the recommended dietary allowance and avoid consuming high doses of any micronutrient.

In particular, vitamin D deficiency might be a concern for individuals with cancer^18^ and has also been studied in healthy elderly populations to prevent or treat loss of MM.^121^, ^122^ Vitamin D combined with whey protein improves muscle function and mass in older adults, either with^121^ or without^122^ exercise. There is a positive association between low vitamin D and loss of MM in individuals >65 years old; in fact, the odds of losing appendicular skeletal MM over 3 years was over two times higher in individuals serum 25‐hydroxyvitamin D ≤50 but >25 nmol/L [odds ratio (OR) 2.25, 95% CI: 1.11, 4.56, *P* < 0.05].^123^ Sufficient vitamin D might also be needed for other supplements to be effective. A trial of older adults individuals randomized to receive a nutritional supplement (HMB, arginine, and lysine) found that while MM increased in treatment and placebo groups, strength only increased in those with sufficient vitamin D levels at baseline (≥30 ng 25OH‐vitamin D_3_/mL).^124^ Considering the current research, vitamin D supplementation according to the recommended dietary allowance of 600–800 international units for individuals with cancer—especially those at risk for poor nutritional status—might be advantageous in the prevention or treatment of low MM.

### Fluid intake

Cancer treatments and side effects may put patients at risk for dehydration,^125^ although the impact of fluid status on MM in this population has not been investigated. Theoretically, low fluid status may exacerbate MM loss because muscular blood flow is reduced during dehydration due to decreased blood pressure and perfusion. Eccentric exercise studies in young, healthy adults suggest that dehydration may increase skeletal muscle damage due to reduced intracellular water, which is hypothesized to induce structural, contractile, and enzymatic protein denaturation.^126^, ^127^ Interestingly, water balance influences protein catabolism in migratory birds during flight^128^, ^129^ and community‐dwelling elderly individuals with low MM have lower mean water intake than individuals with normal levels of MM.^130^ Although further research is needed in this area to support cancer‐specific recommendations, meeting the adequate intake of water (3.7 L/day for adult males and 2.7 L/day for adult females)^131^ may help support protein anabolism in patients with cancer.

### Multimodal interventions

A multimodal intervention consists of two or more modalities aimed at improving specific outcomes. In terms of low MM, exercise combined with nutrition is an especially effective strategy to mitigate muscle loss as it induces net muscle protein anabolism.^70^ Supplements with essential amino acids and carbohydrate consumed after resistance exercise increase muscle protein synthesis rates by 145% above baseline compared with 41% increase from exercise alone in healthy male patients.^132^ An effective dose of protein after exercise is likely around 15–20 g from high‐quality protein sources (i.e. beef, egg, and soy) for young, healthy individuals.^133^ As discussed above, however, individuals with cancer might require more protein to induce anabolism.

Studies in older individuals, obesity, HIV/AIDs, chronic obstructive pulmonary disease, and healthy adults undergoing prolonged bedrest suggest that the combination of nutrition with exercise has the largest impact on MM and strength.^134^ However, a recent systematic review of 37 randomized controlled trials of exercise and nutrition interventions in older adults found that MM, strength, and physical performance improved with exercise in most studies (79.0–92.8%), but an additional effect of nutrition interventions such as protein, essential amino acids, HMB, and creatine was found in a much smaller number of publications (14.3–23.5%).^135^ One possible explanation for the lack of benefit of nutrition interventions include the heterogeneity of study populations in terms of overall health (e.g. bedridden versus ambulatory) and diet; notably, baseline dietary intake of protein was not reported in the studies. The type of exercise training and dietary intervention protocols were also quite different, and the authors noted that nutrition supplementation is more likely to be effective in malnourished individuals. Furthermore, it is possible that nutrient supplementation might displace food consumed in meals, reducing daily intake of the particular nutrient proportional to the amount of the supplementation (i.e. leading a small or null change in nutrient intake).

In cancer, general poor nutritional status and subsequent muscle loss is multifactorial; many patients might start their cancer journey with low MM, suboptimal nutrient intake, and metabolic alterations due to the tumour, and treatment will worsen the condition. Furthermore, median age at diagnosis is 66 years,^136^ and as such, patients with cancer are likely to have multiple co‐morbidities that may exacerbate MM loss. Altered nutrient digestion and absorption (which may be especially pronounced in some cancer types) may also contribute to tissue wasting.^137^ Multimodal interventions may therefore have an additional benefit on improving co‐morbidities and the underlying drivers of muscle catabolism. Our limited knowledge of multimodal therapy effectiveness stems from cancer cachexia, likely due to high inflammation and multi‐faceted nature of this condition.^36^ Two studies in cancer cachexia have established that nutrition modalities such as antioxidant supplementation,^138^, ^139^ l‐carnitine,^139^ or nutritional supplements with EPA^138^ combined with medication such as celecoxib and medroxyprogesterone acetate improve MM. An ongoing international randomized control trial is assessing the impact of nutrition intervention, home‐based exercise, and anti‐inflammatory medication in preventing or attenuating cancer cachexia (Multimodal Exercise/Nutrition/Anti‐inflammatory Treatment for Cachexia Trial [MENAC]).^140^, ^141^ Given the positive findings and theoretical benefit of combining nutrition with other treatments, it is likely that such interventions would be beneficial for individuals with cancer at risk for losing muscle.

## Potential challenges and mitigations of nutrition interventional studies

A common and reasonable question is whether nutrition intervention can impact low MM with lack of a concurrent exercise intervention. Whether nutrition alone can offset a lack of exercise and protect against loss of MM and function in patients with cancer is unknown. We acknowledge that in addition to nutrition, the anabolic environment of muscle is maintained by physical activity and endocrine factors. Specific exercise or hormone/drug interventions should be examined concurrent to nutrition interventions. However, it is important to note that exercise intervention may change nutrient needs. As we expand our understanding on nutritional needs and intervention impact, we can define nutrition and activity interventions that are highly specific and relevant to low MM in oncology patients.

A second concern is the feasibility and impact of nutrition interventions. As previously mentioned, except in the context of refractory cachexia, low MM in cancer is reversible.^13^ Therefore, individuals with cancer have anabolic potential when supported by nutrition interventions.^33^, ^142^, ^143^ Feasibility studies on patients without cachexia are needed and can inform the number of eligible patients, recruitment success, the willingness of participants to be randomized, characteristics of the proposed outcome measure and follow‐up rates and adherence, among others.^144^ Such studies should include follow‐up that is long enough to detect differences in MM or survival.

The impact of cancer‐related and treatment symptoms on the effectiveness of nutrition intervention should also be noted as a concern. Nutritional challenges in working with patients with cancer include possible anorexia or cachexia in response to treatment or tumour progression. As discussed earlier, this can be mitigated by recruiting patients at the time of their diagnoses, which may coincide with the initial stages of disease trajectory. Co‐ordinating assessments within treatment cycles, when symptoms are less severe (e.g. at least 2 weeks after each cycle) may also be helpful. However, in some cancer types, prolonged or unmanageable nutritional impact symptoms may impede or negatively affect oral intake to an extent that jeopardizes the oral nutrition intervention.

As mentioned earlier in this review, the length of intervention is a substantial limitation of the field. Nutritional assessment and modulation should be adopted across the clinical journey (i.e. diagnosis, during and after anti‐cancer treatment), because long‐term adoption of healthy lifestyle patterns are associated with improved outcomes.^67^

Lastly, the generalizability of findings should be considered. Causes of MM loss may differ according to cancer type and/or stage. For example, individuals with head and neck cancer may have severe impairment on their ability to eat, while metastatic cancer is associated with several metabolic changes (i.e. increased systemic inflammation and REE) that negatively impact MM. However, current nutrition guidelines not being specific for cancer type or stage due to the lack of substantial data in homogenous samples of patients. This can also be regarded as a significant limitation of the field; more research on the impact of nutrition interventions on MM according to cancer type and stage is needed.

Although we hereby focus on MM, we acknowledge that adipose tissue is an important component to consider when designing nutrition interventions. We have nonetheless chosen to focus on MM to maintain a reasonable scope of literature. We defer the reader to previous literature reviews on adipose tissue metabolism in cancer.^145^, ^146^

## Conclusions

**Figure 3:**
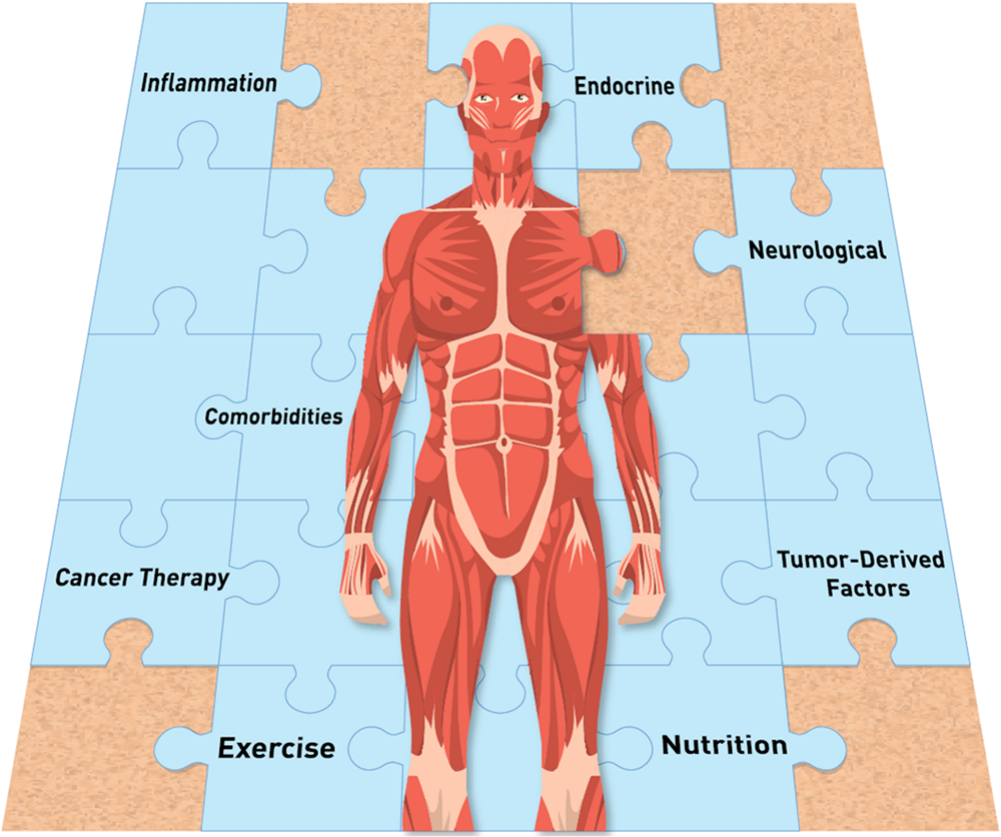
Selected factors impacting body composition in the context of cancer. Nutrition is essential part of supporting optimal muscle mass in these patients.

Low MM is a prevalent and often hidden condition in cancer, which may be neglected by use of anthropometric measurements such as weight, BMI, and weight loss, therefore impacting patient risk stratification. As an important prognostic factor, its prevention and treatment are of upmost importance. Nutrition is an essential piece of the puzzle in supporting adequate MM throughout and beyond cancer disease trajectory (*Figure* 3). Yet targeted nutritional interventions with robust study designs to countermeasure low MM in cancer are needed. These interventions should be administered earlier in the disease trajectory when the window of anabolic potential is open. Further research within this area could directly impact clinical practice and improve cancer care.

## Conflict of Interest

The authors declare that they have no relevant conflicts of interest.
